# A simple, high-throughput, colourimetric, field applicable loop-mediated isothermal amplification (HtLAMP) assay for malaria elimination

**DOI:** 10.1186/s12936-015-0848-3

**Published:** 2015-08-28

**Authors:** Sumudu Britton, Qin Cheng, Colin J. Sutherland, James S. McCarthy

**Affiliations:** University of Queensland, Brisbane, Australia; QIMR Berghofer Medical Research Institute, Brisbane, Australia; Australian Army Malaria Institute, Brisbane, Australia; London School of Hygiene and Tropical Medicine, London, UK

**Keywords:** Loop mediated isothermal amplification, LAMP, Elimination, High throughput, Diagnosis, Malaria

## Abstract

**Background:**

To detect all malaria infections in elimination settings sensitive, high throughput and field deployable diagnostic tools are required. Loop-mediated isothermal amplification (LAMP) represents a possible field-applicable molecular diagnostic tool. However, current LAMP platforms are limited by their capacity for high throughput.

**Methods:**

A high-throughput LAMP (HtLAMP) platform amplifying mitochondrial targets using a 96-well microtitre plate platform, processing 85 samples and 11 controls, using hydroxynaphtholblue as a colourimetric indicator was optimized for the detection of malaria parasites. Objective confirmation of visually detectable colour change results was made using a spectrophotometer. A dilution series of laboratory-cultured 3D7 *Plasmodium falciparum* parasites was used to determine the limit of detection of the HtLAMP assay, using *P. falciparum* (HtLAMP-Pf) and *Plasmodium* genus (HtLAMP-Pg) primers, on whole blood and filter paper, and using different DNA extraction protocols. The diagnostic accuracy of HtLAMP was validated using clinical samples from Papua New Guinea, Malaysia, Ghana and The Gambia and its field applicability was evaluated in Kota Marudu district hospital, Sabah, Malaysia.

**Results:**

The HtLAMP assay proved to be a simple method generating a visually-detectable blue and purple colour change that could be objectively confirmed in a spectrophotometer at a wavelength of 600 nm. When compared with PCR, overall HtLAMP-Pg had a sensitivity of 98 % (n = 260/266, 95 % CI 95–99) and specificity 83 % (n = 15/18, 95 % CI 59–96). HtLAMP-Pf had a sensitivity of 97 % (n = 124/128, 95 % CI 92–99) and specificity of 96 % (n = 151/157, 95 % CI 92–99). A validation study in a regional hospital laboratory demonstrated ease of performance and interpretation of the HtLAMP assay. HtLAMP-Pf performed in this field setting had a sensitivity of 100 % (n = 17/17, 95 % CI 80–100) and specificity of 95 % (n = 123/128, 95 % CI 90–98) compared with multiplex PCR. HtLAMP-Pf also performed well on filter paper samples from asymptomatic Ghanaian children with a sensitivity of 88 % (n = 23/25, 95 % CI 69–97).

**Conclusion:**

This colourimetric HtLAMP assay holds much promise as a field applicable molecular diagnostic tool for the purpose of malaria elimination.

**Electronic supplementary material:**

The online version of this article (doi:10.1186/s12936-015-0848-3) contains supplementary material, which is available to authorized users.

## Background

Malaria elimination is an ambitious but enticing goal. Elimination is defined as “interrupting local mosquito-borne malaria transmission in a defined geographical area, i.e. zero incidence of locally contracted cases, although imported cases will continue to occur” [1]. By definition, malaria elimination is fundamentally different from malaria control, as the focus shifts from detection of symptomatic patients to detection and clearance of all infections. This includes active case detection of asymptomatic persons with sub-patent parasitaemia [2], subjects who have been shown to be able to support ongoing malaria transmission [3].

For *P. falciparum* and *P. vivax,* passive and active detection of infection in the field have relied on two diagnostic tools: thick and thin blood film microscopy, and immunochromatographic detection of circulating parasite antigens using rapid diagnostic tests (RDT). While expert microscopy has been shown to have 100 % sensitivity at thick film parasite densities of 50 parasites/µL, sensitivity decreases significantly under operational field conditions [4]. Furthermore, studies in both Africa and Asia show that microscopy alone will fail to identify a substantial proportion of asymptomatic infections caused by all *Plasmodium* species, compared to detection by PCR [3]. It has been suggested that the proportion of submicroscopic infections is greater in areas where malaria elimination programmes are in place [5]. Such infections remain transmissible, and thus pose a threat to malaria elimination [6]. The success of malaria elimination programs will, therefore, critically depend on successful identification and treatment of all infected individuals. This requires high performance diagnostic tools, as outlined by the malERA consultative group [7]. The target sensitivity has recently been updated by WHO to specify a detection limit of 2 parasites/µL [8], a level substantially lower than expert microscopy.

Loop-mediated isothermal amplification (LAMP) is a nucleic acid detection technology that has the potential to significantly improve malaria field diagnostics, particularly in elimination settings. LAMP differs from PCR in several critical ways. Firstly, it is an isothermal process relying on the *Bacillus stearothermophilus* (Bst) polymerase enzyme. It does not require the cyclical temperature changes [9] necessary for PCR. This facilitates field adaptation of LAMP platforms. Secondly, a positive LAMP reaction results in the formation of a magnesium pyrophosphate precipitate which can be visually detected, by turbidimetry [9] or using metal ion indicators, such as calcein [10], hydroxynaphthol blue [11] and pico-green [12]. LAMP end products have also been visualized using melt curve analysis [13], a bioluminescent output in real time (BART) [14], a lateral flow dipstick [15] and a portable fluorescence detection unit (realAmp) [16].

Since its first description in 2001 [9] LAMP has been applied to the detection of a wide variety of bacterial [17, 18], viral [19], fungal [20] and parasitic [21] organisms, in particular to facilitate diagnosis of infection in resource limited settings. There has been a significant amount of research effort invested into adapting LAMP for diagnosis of malaria. LAMP has been used for the detection of *P. falciparum* using crudely extracted DNA from whole blood [22], or using a rapid boil and spin method [23]. Subsequently, LAMP has been used to identify all Plasmodium species [24], including *P. knowlesi* [25, 26], and LAMP primers have been optimized to improve the sensitivity with which *P. falciparum* [23, 27] and *P. vivax* [28, 29] can be detected. Commercially available Loopamp kits (Eiken Chemical co) have been validated for detection of *P*. *falciparum* [30], including low density infections [31] and for the indirect detection of *P. vivax* using a combination of pan-genus and *P. falciparum*-specific LAMP primers [32]. However, as Hsiang et al. noted, obtaining a high throughput LAMP assay would appear to be an important step in making the technology more widely applicable [33] for the purpose of malaria elimination. Although assay time for most LAMP platforms is rapid at 60–90 min, sample throughput is restricted by the platform used. For example, the LoopAmp kits (Eiken Chemical Co) used in the turbidimeter (Eiken Chemical Co) has a capacity of only 16 samples, while the kits used with a heatblock and ultraviolet lamp has capacity of 46 samples [31]. The demonstration that colorimetric detection of LAMP products provides acceptable diagnostic accuracy [11] suggests that this could be adapted to produce a multi-well, low-cost format for the detection of malaria parasites by LAMP.

This paper describes development of a platform for malaria LAMP that increases the throughput of the assay, and simplifies DNA extraction and sample analysis, resulting in improved field applicability. Validation of the platform was carried out by investigating its diagnostic accuracy using PCR as a gold standard as it is highly sensitive and widely available in reference laboratories for detecting low-level parasitaemia.

## Methods

### Plasmodium parasite and clinical samples

A tenfold dilution series of laboratory-cultured 3D7 *P. falciparum* parasites was prepared in 50 % haematocrit blood using initial samples with parasitaemia adjusted to 1 and 0.5 %, as determined by microscopic examination of thin blood films. The number of parasites per microlitre was calculated using an estimate of 5 × 10^6^ red cells/µL to create a dilution series of samples ranging from 5000 to 0.5 parasites/µL. Four 5 µL blood spots at each dilution were dried for 48 h on blotting paper (Whatman 3MM) before being stored at 4 °C with desiccant. Whole blood was stored at −20 °C.

For assay validation, clinical samples were retrospectively made available from the following clinical trials: (1) filter paper samples (n = 25) from asymptomatic children from Ghana [31, 34], (2) whole blood samples (n = 97) from symptomatic children from The Gambia [35], (3) filter paper samples (n = 42) from symptomatic persons from a high prevalence malaria region in Papua New Guinea (PNG) and (4) filter paper samples (n = 145) from a case–control study [36] undertaken in region of mixed malaria species infections in Sabah, Malaysia. The inclusion and exclusion criteria for each of these studies are outlined in their respective publications.

The study protocols for each study were approved by the Ghana Health Service Ethics Committee (proposal #GHS-ERC-08/7/10) and the Ethics Committee of the London School of Hygiene and Tropical Medicine (proposal #5775), Joint Gambia Government/Medical Research Council Ethics Committee (re. 2002/910), QIMR Berghofer Medical Research Institute Human Research Ethics Committee (P2119), the Malaysian Medical Research Ethics Committee and Menzies School of Health Research, respectively.

### DNA extraction from filter paper

The chelex method for DNA extraction from filter paper [37] was further optimized to improve turnaround time. Briefly, 6 mm filter paper disc punches, corresponding to 5 µL of blood, were incubated in 0.5 % saponin in PBS either overnight (chelex standard protocol) or for 2 h (chelex rapid protocol), centrifuged for 2 min at 4000 rpm, supernatant discarded, washed in PBS, centrifuged for 2 min at 4000 rpm, heated at 98 °C in 150 µL of 6 % chelex (Sigma Aldrich, CAS 8047-15-2) for 30 min and centrifuged for 3 min at 4000 rpm. The resultant 100 µL supernatant was stored at −20 °C.

### DNA extraction from whole blood

For whole blood, the chelex method [38] was further evaluated in two modified protocols. Briefly, 5 µL of whole blood was mixed with either 200 µL of nuclease-free water for 20 min at room temperature (WB-chelex protocol) or 200 µL of 0.5 % saponin, and incubated at 37 °C for 30 min (WB-chelex saponin protocol). Samples were then centrifuged for 3 min at 10,000 rpm, supernatant discarded, the pellet heated at 98 °C in 150 µL of 6 % chelex for 30 min and centrifuged for 3 min at 10,000 rpm. The resultant supernatant was stored at −20 °C. For both extraction protocols, three volumes of blood, 5, 10 and 20 µL, were assessed.

### DNA extraction from clinical samples

The Gambia whole blood samples were extracted using the WB-chelex protocol on 5 µL of blood. The filter paper samples from Ghana were extracted using the FP-chelex standard protocol. The PNG and Sabah filter paper samples were extracted using the FP-chelex rapid protocol. The differences in methods used for clinical sample processing reflect the different sample sets used to validate each of the extraction protocols.

### High-throughput LAMP (HtLAMP) method

**Fig. 1:**
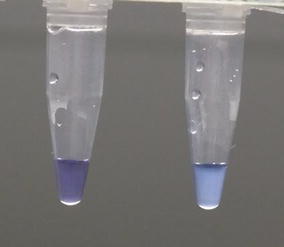
HtLAMP colour change associated with hydroxynaphtholblue (HNB). *Left* clear, *purple colour*, without precipitate = negative. *Right Blue colour* change with cloudy precipitate = positive

High throughput (HtLAMP) was performed in a 96-well microtitre plate as described [11], with some modifications. The assays were performed in a standard u-bottom microtitre plate (Sterihealth) covered with a clear disposable plate cover (Eppendorf). Each plate was able to process 85 samples allowing for 9 negative and 2 positive controls. Reactions were performed in 25 µL total volume containing 1X buffer (20 mM Tris HCL pH 8.8, 10 mM KCl, 8 mM MgSO4, 10 mM NH_4_SO_4_), 1.25 mM each dNTP, 3.55 µM each Pf869FIP/BIP, 1.78 µM each Pf869LF/LBn, 0.2 µM each Pf869F3/B3, 1.18 µM each PgID19FIP/BIP, 0.59 µM each PgID19 LF/LB and 0.15 µM each PgID19 F3/B3, 120 µM Hydroxynaphthol-blue (Fluka, CAS number 63451-35-4) and 8 units Bst polymerase (New England Biolabs, Ipswich, MA, USA). Primer sequences for Pf869 and PgID19 were as published by Polley et al. [23] except Pf869LBn GTTGAGATGGAAACAGCCGG. Microtitre plates were incubated in a water bath at 65 °C for 40 min before the colour change and precipitate in each well were recorded. Blue colour change with a visible precipitate was read as a positive result and purple colour change without a precipitate was read as a negative result (Fig. 1). Positive and negative samples were read by a single person who was blinded to the multiplex PCR results for the samples being analysed.

Performance of HtLAMP in a laboratory setting with limited resources involved storing reagents in a freezer at −20 °C and preparation of the mastermix as required for performance of each HtLAMP assay. Efforts to prevent contamination were based on physical separation of DNA extraction from mastermix preparation areas and spectrophotometry, combined with stringent attention to hand hygiene and glove and gown changes between the different assay areas.

### Limit of detection of HtLAMP

The limit of detection (LOD) for the HtLAMP assay was determined by performing HtLAMP-Pg and HtLAMP-Pf on duplicate samples at each dilution. Filter paper extracted using the two DNA extraction protocols and whole blood 5, 10 and 20 µL extracted using the two DNA extraction protocols were evaluated. The LOD was determined to be the lowest dilution (containing the least parasite material) at which both duplicates were positive by HtLAMP.

### Factors affecting Hydroxynaphthol blue (HNB)

HNB is a metal ion indicator that is sensitive to changes in pH as well as concentrations of the divalent cations magnesium and calcium. Using pH calibration buffers at pH 4.0, 7.0 and 10.0 plus hydrochloric acid, 8 samples of different pH were produced and tested with HNB. Resulting colour changes were recorded visually. Magnesium sulphate (MW 246.68 g/mol) and calcium chloride (MW 110.99 g/mol) were used to make 5 samples of concentrations varying from 1 to 0.001 M. These were also tested with HNB and resulting colour changes were recorded visually.

### Spectrophotometer for determining threshold of positive result

A plate spectrophotometer (PowerwaveXS2 software version 2.00.18) was used in order to determine a threshold value to objectively differentiate a positive from a negative HtLAMP result and demonstrate the reliability of visually detectable colour change in determining a positive and negative sample. Using a *P. falciparum* 3D7 DNA dilution series, HtLAMP mastermix reagents both with and without HNB were placed in the microtitre plate. Nuclease free water was used as the no template negative control. Following incubation in a water bath at 65 °C for 40 min, the microtitre plate was read visually to document cloudy and blue positive reactions and clear and purple negative reactions in the wells containing HNB. The presence and absence of cloudy white precipitate was recorded in the wells without HNB. The microtitre plate was then read in the spectrophotometer across the visible light spectrum from 300 to 700 nm to identify the wavelengths that best discriminated positive and negative samples both with and without HNB.

The optical density (OD) value with the best discrimination of HNB related positive and negative colour change as well as presence and absence of magnesium pyrophosphate precipitate was then used to read each well. The threshold value for a positive reaction was calculated using the mean plus two standard deviations of the no template control (NTC) wells. This threshold value was then subtracted from the OD reading for each well to obtain a positive or negative numerical value. The presence of a visible blue colour change with presence of cloudy precipitate was mandatory for a sample to be positive. A positive numerical OD value was used to confirm the visually determined positive result. A sample that was purple and clear was considered a negative result with a negative OD value confirming this result. Samples with only a positive OD result without corresponding colour change and precipitate were considered negative. Therefore, results were recorded based on their colour change with OD values being used to confirm the visually detected result. Only samples with the appropriate colour change and corresponding OD result were considered true positives or true negatives for the purpose of statistical analysis.

### Nested PCR

For both whole blood and filter paper DNA extraction methods, the limit of detection was determined by comparison of HtLAMP to nested PCR as described by Snounou et al. [39]. PCR reactions were performed in 20 µL total volume containing 1X buffer, 2 mM MgCl2, 200 µM dNTPs, 200 µM primer mix (rPLU5new/rPLU6 for nest 1 and rFAL1/rFAL2 for nest 2) and 0.5 U Taq polymerase (Bioline). The PCR amplification products were visualized by gel electrophoresis.

### Multiplex PCR

PCR was used as the reference standard for analysing the diagnostic accuracy of the HtLAMP platform given its sensitivity. Multiplex PCR (mPCR), which has an LOD of 0.02–0.05 parasites/µL as described by Padley et al. [40], and had already been performed on these samples as part of their respective studies, was used as the gold standard reference test for comparison of HtLAMP-Pf and HtLAMP-Pg in the analysis of the PNG and Sabah samples. Nested PCR [39] was performed on the Ghana and Gambia samples.

### Statistics

Sensitivity and specificity of HtLAMP was calculated against mPCR and nested PCR. Sensitivity = true positives/(true positives + false negatives) and specificity = true negatives/(true negatives + false positives). The 95 % confidence intervals were calculated using an online calculation tool [41].

### Feasibility of field application of htLAMP

The HtLAMP assay field applicability was assessed by performing the assay in a regional hospital in Kota Marudu district in Sabah, Malaysia. DNA was extracted from filter paper samples of 145 symptomatic patients using the FP-chelex rapid protocol, assayed using HtLAMP-Pg and HtLAMP-Pf and resulted were interpreted visually and confirmed with a portable spectrophotometer. Following a 3-day training workshop for local malaria staff on performance and interpretation of HtLAMP assay results, a questionnaire was completed on challenges of performing the HtLAMP assay in a regional laboratory setting.

## Results

### Limits of detection of HtLAMP

The HtLAMP limit of detection was dependent on sample type, i.e. whole blood or filter paper, DNA extraction method and the LAMP primer used (*Plasmodium* genus primers PgID19 or *P. falciparum* primers Pf869 [23]) (Table 1). In the HtLAMP platform, overall, the *Plasmodium* genus LAMP primers (HtLAMP-Pg) performed with a better analytical sensitivity than the *P. falciparum* LAMP primers (HtLAMP-Pf). For whole blood 10 and 20 µL, the chelex-saponin DNA extraction protocol resulted in better analytical sensitivity compared to using chelex alone. The explanation for the discrepancy in results for whole blood 5 µL for the two extraction protocols are unclear but may include pipetting error of blood at small volumes and partial loss of red cell pellet during the DNA extraction process. The best analytical sensitivity for HtLAMP-Pg was achieved using 10 µL of whole blood with the WB-chelex saponin protocol. This had a LOD of 2.5 parasites/µL which was equal to the nested PCR used. The best LOD for HtLAMP-Pf was 5 parasites/µL using 20 µL of whole blood with the WB-chelex saponin protocol. This was equal to the LOD for HtLAMP-Pg at this volume, while nested PCR had an LOD of 0.5 parasite/µL.

**Table 1:** Summary of limits of detection for HtLAMP-Pg and HtLAMP-Pf for whole blood and filter paper with different DNA extraction protocols

Sample (n = 4)	DNA extraction protocol	Limit of detection nested PCR (parasites/µL)	Limit of detection HtLAMP-Pg (parasites/µL)	Limit of detection HtLAMP-Pf (parasites/µL)
Whole blood 5 µL	WB-chelex	5	5	50
	WB-chelex-saponin	25	50	50
Whole blood 10 µL	WB-chelex	2.5	25	50
	WB-chelex-saponin	2.5	2.5	50
Whole blood 20 µL	WB-chelex	25	50	50
	WB-chelex-saponin	0.5	5	5
Filter paper 5 µL	Chelex standard protocol (overnight)	2.5	2.5	5
	Chelex rapid protocol (2 h)	5	25	50

For filter paper, the standard chelex protocol, with an overnight incubation step performed better than the rapid protocol with a 2 h incubation step, providing a tenfold lower (LOD) for HtLAMP-Pg, i.e. 2.5 parasites/µL compared to 25 parasites/µL. For filter paper samples, the LOD for HtLAMP-Pg using the FP-standard chelex protocol for DNA extraction was 2.5 parasites/µL, while the sensitivity of HtLAMP-Pg using the FP-rapid chelex protocol was 25 parasites/µL. The HtLAMP-Pf assay was able to detect 5 parasites/µL using the FP-standard chelex protocol and 50 parasites/µL using the FP-rapid chelex protocol. Therefore, the FP rapid chelex protocol appears to trade-off analytical sensitivity for a faster turnaround time for the assay. Taken together, these results confirm that the starting sample material and method of DNA processing have important effects on the analytical sensitivity of the HtLAMP assay.

In the interest of improving turnaround time of the assay the FP- rapid chelex protocol was chosen despite a compromise in analytical sensitivity for the validation of the HtLAMP platform on filter paper samples from PNG and field testing in Sabah.

### Optimal wavelength for developing an objective threshold for positive results

**Fig. 2:**
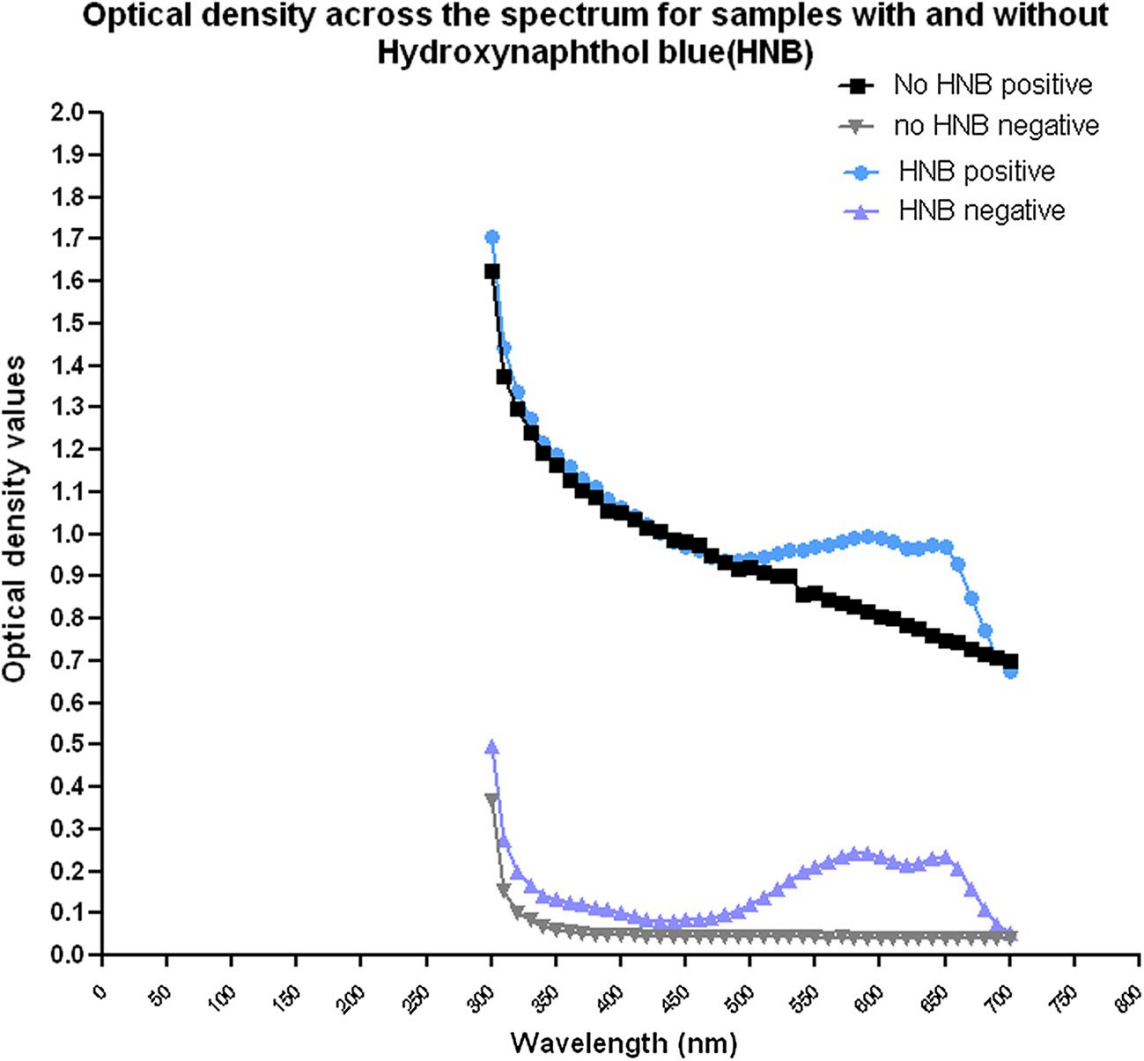
Optical density values across the visible spectrum of light for HtLAMP samples with and without HNB. Between the wavelengths of 550–650 nm higher optical densitometry readings were found for samples that were positive by HtLAMP (no HNB positive and HNB positive). In addition, at these wavelengths, the optical densitometry readings were also able to distinguish samples that were positive due to the presence of the magnesium pyrophosphate precipitate (no HNB positive) and samples that were by positive by HtLAMP due to the blue colour change associated (HNB positive)

When interrogated across the visible spectrum of light, wavelengths between 600 and 650 nm, appeared to best distinguish positive from negative samples, whether the cloudy magnesium pyrophosphate precipitate was present or not and whether there was a blue or purple colour change associated with HNB (Fig. 2). The wavelength of 600 nm was chosen to provide OD values for the HtLAMP assay for the following reasons; (a) this value produced OD values that discriminated well between cloudy and blue positive HtLAMP reactions and clear and purple negative samples in order to calculate an objective OD threshold for each plate of samples and (b) a 600 nm filter is a standard setting for many bench top spectrophotometers. Table 2 shows the correlation between the visually detected colour change and corresponding OD-derived threshold values for the interpretation of HtLAMP-Pf results for a subset of samples from the field validation study in Sabah.

**Table 2:**
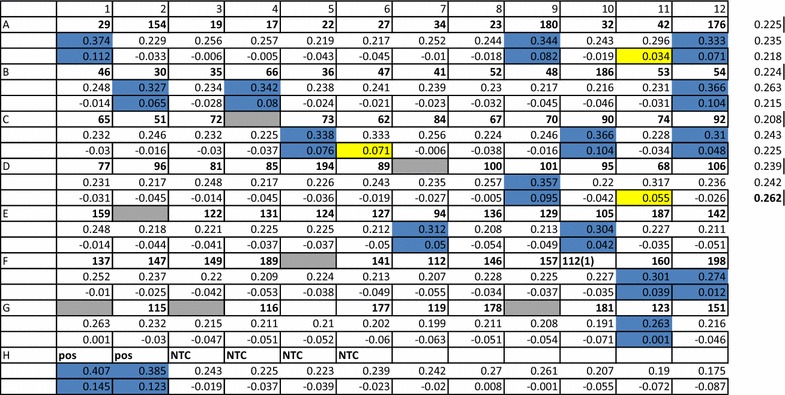
HtLAMP-Pf optical densitometry (OD) results for 77 filter paper samples demonstrating the correlation between the visually detected colour change results for positive samples and their correlation with the positive results based on an OD-derived threshold value

Samples in blue had a blue/cloudy colour change as well as a corresponding positive OD threshold value. Samples in yellow were positive only by OD threshold. Shaded grey wells were negative controls within the plate. OD values for all negative control samples in each plate are used to calculate the OD threshold value (mean plus two standard deviations) which in this example is 0.262

### Effect of pH, magnesium sulphate and calcium chloride on HNB colour change

The colour change associated with HNB was significantly affected by changes in pH, magnesium and calcium. At pH 3, HNB had a pink appearance compared with dark blue at pH 7 and sky blue at pH 10. Colour variation was also seen with magnesium, ranging from pink at a concentration of 0.001 M concentration to dark purple at a concentration of 1.0 M, and calcium, from dark pink at 0.01 M concentration to bright blue at 0.5 M concentrations. This suggests that maintaining the correct concentration of calcium, magnesium and pH of the mastermix, as well as awareness of other metal ions present during extractions processes, is important in allowing HNB to undergo its colour change during a LAMP reaction.

### Sensitivity and specificity of HtLAMP-Pg and HtLAMP-Pf in clinical samples from symptomatic patients for the detection of *P. falciparum*

Filter paper blood spots from symptomatic patients from Papua New Guinea and Sabah, Malaysia and whole blood samples from The Gambia were evaluated using HtLAMP-Pg and HtLAMP-Pf to determine the diagnostic accuracy of the assay for the detection of *P. falciparum*. The features of each of these sample sets and composite sensitivity and specificity of HtLAMP-Pg/Pf for the detection of *P. falciparum* are outlined in Table 3.

**Table 3:** Sensitivity and specificity of HtLAMP-Pg/Pf for the detection of *P. falciparum*

Origin	Assay	Diagnostic accuracy		No. of samples	Type of sample	PCR technique used for speciation	Pf	Pv	Pm	Pk	Po spp	Mixed infections	Negatives
Papua New Guinea				42	Filter paper	Multiplex PCR	12	9	0	0	0	5 (Pf/Pv)	16
	HtLAMP-Pg	Sensitivity	77 % (20/26) 95 % CI 56–91										
		Specificity	81 % (13/16) 95 % CI 54–96										
	HtLAMP-Pf	Sensitivity	76 % (13/17) 95 % CI 50–93										
		Specificity	100 % (25/25) 95 % CI 86–100										
Sabah, Malaysia				145	Filter paper	Multiplex PCR and nested PCR (Pk)	17	64	7	56	0	1 (Pv/Pk)	0
	HtLAMP-Pg	Sensitivity	100 % (145/145) 95 % CI 97–100										
	HtLAMP-Pf	Sensitivity	100 % (17/17) 95 % CI 80–100										
		Specificity	95 % (123/128) 95 % CI 90–98										
The Gambia				97	Whole blood	Nested PCR	94	NA	1	NA	0	0	2
	HtLAMP-Pg	Sensitivity	100 % (95/95) 95 % CI 96–100										
		Specificity	100 % (2/2) 95 % CI 16–100										
	HtLAMP-Pf	Sensitivity	100 % (94/94) 95 % CI 96–100										
		Specificity	100 % (3/3) 95 % CI 29–100										
TOTAL				284			124	73	8	56	0	6	18
	HtLAMP-Pg	Sensitivity	98 % (260/266) 95 % CI 95–99										
		Specificity	83 % (15/18), 95 % CI 59–96										
		PPV	99 %, 95 % CI 97–100										
		NPV	71 %, 95 % CI 48–89										
	HtLAMP-Pf	Sensitivity	97 % (124/128) 95 % CI 92–99										
		Specificity	96 % (151/157) 95 % CI 92–99										
		PPV	95 %, 95 % CI 90–98										
		NPV	97 %, 95 % CI 94–99										

This table summarizes the features of each of the 3 sample sets, from Papua New Guinea, Sabah Malaysia and The Gambia, and a composite sensitivity and specificity of HtLAMP-Pg/Pf for the detection of *P. falciparum*

Overall, HtLAMP-Pg had a sensitivity of 98 % (n = 260/266, 95 % CI 95–99) and specificity of 83 % (n = 15/18, 95 % CI 59–96) for the detection of *Plasmodium* species. HtLAMP-Pf had a sensitivity of 97 % (n = 124/128, 95 % CI 92–99) and specificity of 96 % (n = 151/157, 95 % CI 92–99) for the detection of *P. falciparum.*

### Sensitivity and specificity of the combination of HtLAMP-Pg and HtLAMP-Pf for the detection of non-falciparum *Plasmodium* mono-infection in symptomatic patients

The diagnostic accuracy of the combination of HtLAMP-Pg and HtLAMP-Pf for the detection of non-falciparum *Plasmodium* species mono-infections was also assessed. In this context, the sensitivity of HtLAMP-Pg was 100 % (n = 232/232, 95 % CI 98–100), with a specificity of 83 % (n = 15/18, 95 % CI 56–96).

Subgroup analysis demonstrated that in the Papua New Guinea samples, HtLAMP-Pg had a sensitivity of 100 % (n = 9/9, *P. vivax* positive) and specificity of 81 % (n = 13/16). There were 3 false positive HtLAMP-Pg results. HtLAMP-Pf was negative in all the mono-infection samples. For the 128 non-*falciparum* species infections from Sabah, HtLAMP-Pg had a sensitivity of 100 % (n = 128/128). HtLAMP-Pf was negative for 122 of these samples. For the 6 false positive HtLAMP-Pf results (five *P. vivax*, one *P. knowlesi*) there was no evidence of *P. falciparum* co-infection by PCR. In the Gambia samples, of the 95 HtLAMP-Pg positive samples, only 1 was negative by HtLAMP-Pf, suggesting a non-*falciparum* species mono-infection which was confirmed by PCR to be *P. malariae* with a parasitaemia of 7.5 parasites/µL.

### Sensitivity of HtLAMP in persons with asymptomatic parasitaemia

Features of the 25 samples chosen from a set of samples from asymptomatic school children in Kumasi, Ghana are outlined (see Additional file 1). Of the 25 samples 100 % were positive for *P. falciparum* by nested PCR and 44 % were co-infected with *P. malariae*. Interestingly, HtLAMP-Pg was positive for two samples in which only *P. falciparum* gametocytes were seen on microscopy of which one was also positive by HtLAMP-Pf. Compared with nested PCR the sensitivity of HtLAMP-Pg was 96 % (n = 24/25, 95 % CI 80–100) and sensitivity of HtLAMP-Pf was 88 % (n = 22/25, 95 % CI 69–97) (Table 4). HtLAMP-Pg and HtLAMP-Pf were able to detect the samples with the lowest parasitaemia of 40 parasites/µL (n = 3).

**Table 4:** Sensitivity and specificity of HtLAMP compared with nested PCR in Ghanaian children with asymptomatic parasitaemia

	Sensitivity	PPV
HtLAMP-Pg (compared with nested PCR)	96 % (24/25) 95 % CI 80–100	100 % 95 % CI 85–100
HtLAMP-Pf (compared with nested PCR)	88 % (22/25) 95 % CI 69–97	100 % 95 % CI 85–100

### Feasibility of field application of HtLAMP

HtLAMP was performed in a regional hospital laboratory in Kota Marudu district, Sabah, Malaysian Borneo. This laboratory was able to perform basic haematology and biochemistry testing but did not perform microbiological culture or molecular assays. The HtLAMP assay was performed in a plastic bucket adapted to function as a water bath using a heating element and thermometer. A portable spectrophotometer (ChroMate) with a 600 nm filter provided the optical densitometry values. Samples and HtLAMP reagents were stored at −20 °C in a freezer. Three separate benches within a large room were dedicated to DNA extraction, HtLAMP assay setup and spectrophotometry in order to prevent cross-contamination.

The HtLAMP-Pg assay, performed in this resource-limited laboratory setting, had a sensitivity of 100 % (n = 145/145, 95 % CI 97–100). The HtLAMP-Pf assay had a sensitivity of 100 % (n = 17/17, 95 % CI 80–100) and a specificity of 95 % (n = 123/128, 95 % CI 90–98).

Two local malaria laboratory staff was trained over a 3 day period to perform DNA extraction from filter paper and whole blood, setup the assay and interpret the results of the HtLAMP assay. Issues that were identified by local staff as potential limitations to deployment of HtLAMP in a field laboratory were difficulty of accurate sample placement into the 96-well plate and the availability and cost of reagents. However, it was felt that these limitations were balanced against the rapid turnaround time for the detection of parasites in microscopy negative samples thereby facilitating earlier patient treatment and household screening, potential time and cost saving in avoiding sending samples for PCR testing at reference laboratories and ease of interpretation of visual colour change results on the HtLAMP assay. The main challenges to setting up HtLAMP in this non-reference, low resource setting laboratory were ensuring good workflow to prevent contamination, and ensuring the reliability of ancillary equipment such as the centrifuge and freezers.

## Discussion

To achieve its ambitious goals, the malaria elimination agenda will likely require diagnostic capacity to actively identify sub-patent infection in order to interrupt transmission. PCR, the most sensitive molecular diagnostic tool currently available, is limited by its technical requirements, particularly in resource limited field settings. A robust, low-cost and high-throughput assay for the rapid detection of malaria parasite DNA in finger-prick blood samples would provide this capacity. LAMP has been adapted into several different platforms [16, 31, 42–44] in order to improve its field applicability and throughput. While the definition of the throughput required for the application of LAMP for the purpose of malaria elimination remains to be established, the number of samples able to be processed simultaneously on these platforms range from 5 [42] to 46 [31, 44] per assay.

The HtLAMP assay for the diagnosis of malaria that is described here has a simple platform—a standard 96-well microtitre plate, covered with a clear plate seal and incubated in a water bath at 65 °C, that is able to process a minimum of 85 samples (allowing for positive and negative controls). The use of a colorimetric indicator enhances visual detection of the assay endpoint. To improve objectivity of interpretation of results, the assay uses a spectrophotometer at 600 nm to produce a threshold value which correlates with the visible colour change. In addition, DNA extraction protocols for whole blood and filter paper based on chelex and saponin have been modified to improve turnaround time while maintaining the cost effectiveness of the assay.

However, there appears to be significant variation in the LOD of HtLAMP depending on sample type, DNA extraction method and primers selected, particularly so for filter paper samples where there was a ten-fold loss of analytical sensitivity using a DNA extraction method that had a faster turnaround time. Nevertheless, HtLAMP-Pg using 10 µL of whole blood with a chelex saponin protocol and HtLAMP-Pg on a 5 µL filter paper blood spot using the chelex overnight protocol achieved a LOD of 2.5 parasites/µL. This suggests that the HtLAMP platform has the potential to reach the WHO target LOD. However, further modifications, such as using commercial kits, optimisation of low cost DNA extraction methods or using a larger volume of blood are likely to be required to achieve the balance between improved analytical sensitivity and turnaround time. The availability of more sensitive LAMP primers to detect *Plasmodium* genus and species- specific primers may also enhance the LOD of the HtLAMP assay. Therefore, malaria elimination programs choosing diagnostic tools may be required to make important operational and logistics decisions with regards to both the type of assay used and the sample types, for example whole blood or filter paper, that can achieve the best analytical sensitivity for that assay in order to meet the current WHO target of 2 parasites/µL.

The HtLAMP assay has been validated in a resource limited laboratory setting using filter paper samples and the chelex rapid DNA extraction protocol, despite its aforementioned limitations, in order to evaluate its turnaround time in this clinical setting. Compared with PCR, which requires samples to be sent to a reference laboratory, HtLAMP-Pg had a sensitivity of 100 % and HtLAMP-Pf had a sensitivity of 100 % and a specificity of 95 % and results were available within 24 h.

In this study, the HtLAMP assay was validated for the detection of *Plasmodium* genus and *P. falciparum* on clinical samples from microscopy positive symptomatic and asymptomatic patients from regions with different *Plasmodium* species prevalence. Overall, HtLAMP-Pg had a sensitivity of 98 % and specificity of 83 % and HtLAMP-Pf had a sensitivity of 97 % and specificity of 96 % for the detection of *P. falciparum.* The use of the combination of *Plasmodium* genus and *P. falciparum* LAMP primers showed a HtLAMP-Pg sensitivity of 100 % and specificity 83 % compared to PCR for the detection of non-falciparum *Plasmodium* monoinfections. These results are comparable to other LAMP studies in symptomatic patients (Table 5). However, further studies are required to determine the sensitivity and specificity of HtLAMP in detecting sub-microscopic infections in asymptomatic patients.

**Table 5:** Comparison of sensitivity and specificity of HtLAMP-Pg and HtLAMP-Pf for the detection of *P. falciparum* and non-*falciparum* species with other LAMP studies in symptomatic patients

LAMP study	Sample source	Species	LAMP primer	Sensitivity (%)	Specificity (%)
HtLAMP	Symptomatic patients	*P. falciparum*	Pg	98	83
			Pf	97	96
Polley et al. [23]	Symptomatic patients	*P. falciparum*	Pg	93.9	100
			Pf	93.3	100
Polley et al. [27]	Symptomatic returned travellers	*P. falciparum*	Pg	97	99.2
			Pf	98.4	98.1
Hopkins et al. [30]	Passive case detection	*P. falciparum*	Pf	89–93.9	76.3–85
HtLAMP	Symptomatic patients	Non-*falciparum*	Pg	100	83
Vallejo et al. [32]	Symptomatic patients	Non-*falciparum*	Pg	91.4	91.8

The 96 well HtLAMP platform is able to test 85 samples per plate, allowing for positive and negative controls. The cost per test in this platform is USD$ 0.85 excluding primers and DNA extraction. Final cost will depend on the starting material (filter paper or whole blood), the use of either crude or commercial DNA extraction protocols and the primer sets selected. Nevertheless, the cost per sample to perform HtLAMP-Pg on whole blood extracted using chelex-saponin protocol is USD$1. The turnaround time for completion of an assay, including DNA extraction, is 4–6 h.

However, HtLAMP is not a quantitative assay, a limitation it shares with other LAMP based assays. Other potential limitations include the use of HNB, which is sensitive to changes in pH and metal ion concentrations. Awareness of, for example, the type of DNA extraction method used on samples, which may contain reagents that affect pH and metal ion concentrations, would help maintain the reliability of interpretation of the colour change aspect of the assay. Nevertheless, this is balanced against its low cost, stability at room temperature and easy visual interpretation of results. In its current format, HtLAMP relies on the storage of reagents at −20 °C. While an important issue in terms of field deployment, this could potentially be overcome by lyophilization of reagents.

## Conclusion

The HtLAMP platform offers several important improvements over currently available LAMP platforms. Firstly, HtLAMP is able to process up to 85 samples simultaneously which makes it an ideal potential platform for surveillance or household surveys. Secondly, it uses standard consumables, and can be performed using DNA extracted by chelex and saponin based protocols which makes it very cost effective. Thirdly, one of the significant advantages to the use of HNB is that it can be added to the start of the assay such that HtLAMP remains a closed system, thereby reducing the risk of cross-contamination. Fourthly, while the platform of HtLAMP has been used here with *Plasmodium* genus and *P. falciparum* primers, it could be adapted to detect any other blood-borne pathogen of interest, thereby broadening the potential applications of the platform beyond the diagnosis of malaria. Finally, HtLAMP has been performed successfully in a regional laboratory setting by local staff trained over a very short period of time demonstrating its ease of performance and interpretation of results.

This provides confidence that the HtLAMP could be usefully deployed as a molecular diagnostic tool to support malaria elimination in resource-limited settings.

## Additional file

Additional file 1: Features of asymptomatic Ghanaian children samples (n=25). Table outlines features of the 25 samples chosen from a set of samples from asymptomatic school children in Kumasi, Ghana.

## References

[1] WHO (2008). Global malaria control and elimination: report of a technical review.

[2] WHO (2012). Disease surveillance for malaria elimination: an operational manual.

[3] Okell LC, Bousema T, Griffin JT, Ouedraogo AL, Ghani AC, Drakeley CJ (2012). Factors determining the occurrence of submicroscopic malaria infections and their relevance for control. Nat Commun.

[4] Coleman RE, Maneechai N, Rachaphaew N, Kumpitak C, Miller RS, Soyseng V (2002). Comparison of field and expert laboratory microscopy for active surveillance for asymptomatic *Plasmodium falciparum* and *Plasmodium vivax* in western Thailand. Am J Trop Med Hyg.

[5] Okell LC, Ghani AC, Lyons E, Drakeley CJ (2009). Submicroscopic infection in *Plasmodium falciparum*-endemic populations: a systematic review and meta-analysis. J Infect Dis.

[6] Bousema T, Drakeley C (2011). Epidemiology and infectivity of *Plasmodium falciparum* and *Plasmodium vivax* gametocytes in relation to malaria control and elimination. Clin Microbiol Rev.

[7] MalERA Consultative Group on Diagnoses and Diagnostics. A research agenda for malaria eradication: diagnoses and diagnostics. PLoS Med 2011; 8:e1000396. 10.1371/journal.pmed.1000396 PMC3026696 21311583

[8] WHO (2014). WHO evidence review group on malaria diagnosis in low transmission settings, 16–18 December 2013.

[9] Mori Y, Nagamine K, Tomita N, Notomi T (2001). Detection of loop-mediated isothermal amplification reaction by turbidity derived from magnesium pyrophosphate formation. Biochem Biophys Res Commun.

[10] Tomita N, Mori Y, Kanda H, Notomi T (2008). Loop-mediated isothermal amplification (LAMP) of gene sequences and simple visual detection of products. Nat Protoc.

[11] Goto M, Honda E, Ogura A, Nomoto A, Hanaki K (2009). Colorimetric detection of loop-mediated isothermal amplification reaction by using hydroxy naphthol blue. Biotechniques.

[12] Wastling SL, Picozzi K, Kakembo ASL, Welburn SC (2010). LAMP for Human African trypanosomiasis: a comparative study of detection formats. PLoS Negl Trop Dis.

[13] Yamamura M, Makimura K, Ota Y (2009). Evaluation of a new rapid molecular diagnostic system for *Plasmodium falciparum* combined with DNA filter paper, loop-mediated isothermal amplification, and melting curve analysis. Jpn J Infect Dis.

[14] Kiddle G, Hardinge P, Buttigieg N, Gandelman O, Pereira C, McElgunn CJ (2012). GMO detection using a bioluminescent real time reporter (BART) of loop mediated isothermal amplification (LAMP) suitable for field use. BMC Biotechnol.

[15] Njiru ZK, Mikosza AS, Armstrong T, Enyaru JC, Ndung’u JM, Thompson AR (2008). Loop-mediated isothermal amplification (LAMP) method for rapid detection of *Trypanosoma brucei* rhodesiense. PLoS Negl Trop Dis.

[16] Lucchi NW, Demas A, Narayanan J, Sumari D, Kabanywanyi A, Kachur SP (2010). Real-time fluorescence loop mediated isothermal amplification for the diagnosis of malaria. PLoS One.

[17] Ou X, Li Q, Xia H, Pang Y, Wang S, Zhao B (2014). Diagnostic accuracy of the PURE-LAMP test for pulmonary tuberculosis at the county-level laboratory in China. PLoS One.

[18] Dittrich S, Castonguay-Vanier J, Moore CE, Thongyoo N, Newton PN, Paris DH (2014). Loop-mediated isothermal amplification for *Rickettsia typhi* (the causal agent of murine typhus): problems with diagnosis at the limit of detection. J Clin Microbiol.

[19] Luo L, Nie K, Yang MJ, Wang M, Li J, Zhang C (2011). Visual detection of high-risk human papillomavirus genotypes 16, 18, 45, 52, and 58 by loop-mediated isothermal amplification with hydroxynaphthol blue dye. J Clin Microbiol.

[20] Scheel CM, Zhou Y, Theodoro RC, Abrams B, Balajee SA, Litvintseva AP (2014). Development of a loop-mediated isothermal amplification method for detection of *Histoplasma capsulatum* DNA in clinical samples. J Clin Microbiol.

[21] Arimatsu Y, Kaewkes S, Laha T, Sripa B (2015). Specific diagnosis of *Opisthorchis viverrini* using loop-mediated isothermal amplification (LAMP) targeting parasite microsatellites. Acta Trop.

[22] Poon LL, Wong BW, Ma EH, Chan KH, Chow LM, Abeyewickreme W (2006). Sensitive and inexpensive molecular test for falciparum malaria: detecting *Plasmodium falciparum* DNA directly from heat-treated blood by loop-mediated isothermal amplification. Clin Chem.

[23] Polley SD, Mori Y, Watson J, Perkins MD, Gonzalez IJ, Notomi T (2010). Mitochondrial DNA targets increase sensitivity of malaria detection using loop-mediated isothermal amplification. J Clin Microbiol.

[24] Han E, Watanabe R, Sattabongkot J, Khuntirat B, Sirichaisinthop J, Iriko H (2007). Detection of four Plasmodium species by genus and species-specific loop mediated isothermal amplication for clinical diagnosis. J Clin Microbiol.

[25] Lau YL, Fong MY, Mahmud R, Chang PY, Palaeya V, Cheong FW (2011). Specific, sensitive and rapid detection of human *Plasmodium knowlesi* infection by loop-mediated isothermal amplification (LAMP) in blood samples. Malar J.

[26] Iseki H, Kawai S, Takahashi N, Hirai M, Tanabe K, Yokoyama N (2010). Evaluation of a loop-mediated isothermal amplification method as a tool for diagnosis of infection by the zoonotic simian malaria parasite *Plasmodium knowlesi*. J Clin Microbiol.

[27] Polley SD, Gonzalez IJ, Mohamed D, Daly R, Bowers K, Watson J (2013). Clinical evaluation of a loop-mediated amplification kit for diagnosis of imported malaria. J Infect Dis.

[28] Patel JC, Oberstaller J, Xayavong M, Narayanan J, Debarry JD, Srinivasamoorthy G (2013). Real-time loop-mediated isothermal amplification (ReaLamp) for the species-specific identification of *Plasmodium vivax*. PLoS One.

[29] Lu F, Gao Q, Zhou H, Cao J, Wang W, Lim CS (2011). Molecular test for vivax malaria with loop-mediated isothermal amplification method in central China. Parasitol Res.

[30] Hopkins H, Gonzalez IJ, Polley SD, Angutoko P, Ategeka J, Asiimwe C (2013). Highly sensitive detection of malaria parasitemia in a malaria-endemic setting: performance of a new loop-mediated isothermal amplification kit in a remote clinic in Uganda. J Infect Dis.

[31] Cook J, Aydin-Schmidt B, Gonzalez IJ, Bell D, Edlund E, Nassor MH (2015). Loop-mediated isothermal amplification (LAMP) for point-of-care detection of asymptomatic low-density malaria parasite carriers in Zanzibar. Malar J.

[32] Vallejo AF, Martinez NL, Gonzalez IJ, Arevalo-Herrera M, Herrera S (2015). Evaluation of the loop mediated isothermal DNA amplification (LAMP) kit for malaria diagnosis in *P. vivax* endemic settings of Colombia. PLoS Negl Trop Dis.

[33] Hsiang MS, Greenhouse B, Rosenthal PJ (2014). Point of care testing for malaria using LAMP, loop mediated isothermal amplification. J Infect Dis.

[34] Dinko B, Oguike M, Larbi J, Bousema T, Sutherland C (2013). Persistent detection of *Plasmodium falciparum*, *P. malariae*, *P. ovale**curtisi* and *P. ovale wallikeri* after ACT treatment of asymptomatic Ghanaian school-children. Int J Parasitol Drugs Drug Resist.

[35] Sutherland CJ, Ord R, Dunyo S, Jawara M, Drakeley CJ, Alexander N (2005). Reduction of malaria transmission to Anopheles mosquitoes with a six-dose regimen of co-artemether. PLoS Med.

[36] Grigg MJ, William T, Drakeley CJ, Jelip J, von Seidlein L, Barber BE (2014). Factors that are associated with the risk of acquiring *Plasmodium knowlesi* malaria in Sabah, Malaysia: a case-control study protocol. BMJ Open.

[37] Plowe CV, Djimde A, Bouare M, Doumbo O, Wellems TE (1995). Pyrimethamine and proguanil resistance-conferring mutations in *Plasmodium falciparum* dihydrofolate reductase: polymerase chain reaction methods for surveillance in Africa. Am J Trop Med Hyg.

[38] Walsh PS, Metzger DA, Higuchi R (1991). Chelex 100 as a medium for simple extraction of DNA for PCR-based typing from forensic material. Biotechniques.

[39] Snounou G, Viriyakosol S, Zhu XP, Jarra W, Pinheiro L, do Rosario VE (1993). High sensitivity of detection of human malaria parasites by the use of nested polymerase chain reaction. Mol Biochem Parasitol.

[40] Padley D, Moody AH, Chiodini PL, Saldanha J (2003). Use of a rapid, single-round, multiplex PCR to detect malarial parasites and identify the species present. Ann Trop Med Parasitol.

[41] MedCalc. http://www.medcalc.org/calc/diagnostic_test.php.

[42] Sema M, Alemu A, Bayih A, Getie S, Getnet G, Guelig D (2015). Evaluation of non-instrumented nucleic acid amplification by loop-mediated isothermal amplification (NINA-LAMP) for the diagnosis of malaria in Northwest Ethiopia. Malar J.

[43] Yongkiettrakul S, Jaroenram W, Arunrut N, Chareanchim W, Pannengpetch S, Suebsing R (2014). Application of loop-mediated isothermal amplification assay combined with lateral flow dipstick for detection of *Plasmodium falciparum* and *Plasmodium vivax*. Parasitol Int.

[44] Morris U, Khamis M, Aydin-Schmidt B, Abass AK, Msellem MI, Nassor MH (2015). Field deployment of loop-mediated isothermal amplification for centralized mass-screening of asymptomatic malaria in Zanzibar: a pre-elimination setting. Malar J.

