# Supplementary material for: A simple, high-throughput, colourimetric, field applicable loop-mediated isothermal amplification (HtLAMP) assay for malaria elimination
Source: Malar J. 2015 Aug 28;14:335. doi: 10.1186/s12936-015-0848-3 (PMC4552465; doi:10.1186/s12936-015-0848-3)
Supplement: Additional file 1: — Features of asymptomatic Ghanaian children samples (n=25). Table outlines features of the 25 samples chosen from a set of samples from asymptomatic school children in Kumasi, Ghana. [file 12936_2015_848_MOESM1_ESM.doc]

**Additional Data**

**Additional File 1: Features of asymptomatic Ghanaian children samples (n=25)**

| **Features** | **Details** | **Numbers** |
| --- | --- | --- |
| Age groups | Less than 10 years old  10-16 years old  16 years and older | 10  10  5 |
| Microscopy results | Asexual *P. falciparum* only  Asexual *P. falciparum* and gametocytes  *P. falciparum* gametocytes only  *P. malariae* parasites | 21  2  2  0 |
| Parasitemia by microscopy | Range  Number of samples with parasitemia <100 parasites /µL | 40- 5120 parasites /µL  4 |
| Nested PCR results | *P. falciparum* only (Pf)  *P. malariae* only (Pm)  *Pf/ Pm* co-infection | 14  0  11 |
